# The Specialist’s Point of View

**Published:** 1906-08

**Authors:** Ross P. Cox

**Affiliations:** Rome, Ga.


### THE SPECIALIST'S POINT OF VIEW.

I know you've got me on the list,
I fear I can not now be missed,

But I shall have recourse to rhyme
"To make the punishment fit the crime."

I fear I shall in measures sing
That little wit or wisdom bring.

But you can't dream, nor know nor think
The joy that wells up o'er the brink,

To have you here, a score in number,
That dare not groan nor swear nor slumber.

Tho' greatly bored, you're too refined
And too polite to speak your mind.

And tho' my viewpoint well may seem
To touch a somewhat tedious theme,

Some preachments I must perpetrate
As sure as taxes, death or fate.

Here's at you, then, according to
A specialist's own point of view.

Each eye and nose and throat and ear
Is in my own especial sphere;

Oh, never let your greed of pelf
Lead you to treat them by yourself,

Unless you're sure you're right, for then
None should forbid that you wade in.

What tho' mistakes may unseen lie
To mortal brain or mortal eye,

Sometimes a doctor filches mine
And sins some sins along this line.

But on the whole, I must admit,
I've no complaint to make of it.

Great Atropine! that in thy place
Art child of heaven, full of grace:—

In hardened eyes, a demon fell
As any creature spawned of hell.

The imp of eye-strain's protean ills,
Full many a cup of woe it fills:

Of all its evils manifold
The half has never yet been told.

The open mouth, the vacant stare,
The frequent cough, the failing ear,

That make the stupid "tonsil face";
Yet some neglect the days of grace.

Of all the sins that lead to grief,
'Wait to outgrow it" is the chief.

All these, and more, let us forgive
And bid the sinner hope and live;

But there is one I swear that never,
Not in this life nor the forever,

Should gleam of mercy, hope or grace,
E'er find, should one, in any case

Of laryngeal croup refuse,
At need, the lymph and tubes to use,

Lest livid lips and upturned eyes
And choking infants' smothered cries

Haunt all their days until they know
No rest or solace here below.

In every township on the map,
From Tybee light to Rabun gap,

I'd like to swear this gospel truth
That they are murderers in sooth,

Who, fighting croup, *postulo rerum,*
Fail to make use of tubes and serum.

But sins like these are rare, with us,
Physicians conscientious.

Leastwise, 'tis pleasanter to view
The many merits than "to chew

The cud of bitter memories,"
When we might find so much to praise.

Now further, in my point of view,
We each should be more kind and true:

Let deeds of love adorn each day,
We ne'er again shall pass this way.

Than wealth or life hold honor dear,
Without reproach and without fear.

Aim sterling character to gain,
All else is empty, fleeting, vain.

Here's to your larger happiness,
The things that make for true success!

Ross P. Cox.

---

### THE PROFESSION OF MEDICINE.

President Faunce, of Brown University, recently delivered an address before the Rhode Island Medical Society, from which we quote as follows:

"In two respects the medical profession deserves the grateful recogniton and regard of all other callings in modern life. It has always insisted that the practice of medicine is a profession and not a trade. Trade is occupation for livelihood; profession is occupation for the service of the world. Trade is occupation for joy of the result; profession is occupation for joy in the process. Trade is occupation where anybody may enter; profession is occupation where only those who are prepared may enter. Trade is occupation taken up temporarily, until something better offers; profession is occupation with which one is identified for life. Trade makes one the rival of every other trader; profession makes one the cooperator with all his colleagues. Trade knows only the ethics of success; profession is bound by lasting ties of sacred honor."—*Medical Review of Reviews.*

